# Usefulness of melatonin as complementary to chemotherapeutic agents at different stages of the angiogenic process

**DOI:** 10.1038/s41598-020-61622-x

**Published:** 2020-03-16

**Authors:** Alicia González-González, Alicia González, Noemi Rueda, Carolina Alonso-González, Javier Menéndez Menéndez, Carlos Martínez-Campa, Stefania Mitola, Samuel Cos

**Affiliations:** 1 Department of Physiology and Pharmacology, School of Medicine, University of Cantabria and Instituto de Investigación Sanitaria Valdecilla (IDIVAL), 39011 Santander, Spain; 2 Department of Molecular and Translational Medicine, Laboratory for Preventive and Personalized Medicine, University of Brescia, 25123 Brescia, Italy

**Keywords:** Physiology, Breast cancer

## Abstract

Chemotherapeutics are sometimes administered with drugs, like antiangiogenic compounds, to increase their effectiveness. Melatonin exerts antitumoral actions through antiangiogenic actions. We studied if melatonin regulates the response of HUVECs to chemotherapeutics (docetaxel and vinorelbine). The inhibition that these agents exert on some of the processes involved in angiogenesis, such as, cell proliferation, migratory capacity or vessel formation, was enhanced by melatonin. Regarding to estrogen biosynthesis, melatonin impeded the negative effect of vinorelbine, by decreasing the activity and expression of aromatase and sulfatase. Docetaxel and vinorelbine increased the expression of *VEGF-A*, *VEGF-B*, *VEGF-C*, *VEGFR-1*, *VEGFR-3*, *ANG1* and/or *ANG-2* and melatonin inhibited these actions. Besides, melatonin prevented the positive actions that docetaxel exerts on the expression of other factors related to angiogenesis like *JAG1*, *ANPEP*, *IGF-1*, *CXCL6*, *AKT1*, *ERK1*, *ERK2*, *MMP14* and *NOS3* and neutralized the stimulating actions of vinorelbine on the expression of *FIGF*, *FGFR3*, *CXCL6*, *CCL2*, *ERK1*, *ERK2*, *AKT1*, *NOS3* and *MMP14*. In CAM assay melatonin inhibited new vascularization in combination with chemotherapeutics. Melatonin further enhanced the chemotherapeutics-induced inhibition of p-AKT and p-ERK and neutralized the chemotherapeutics-caused stimulatory effect on HUVECs permeability by modifying the distribution of VE cadherin. Our results confirm that melatonin blocks proangiogenic and potentiates antiangiogenic effects induced by docetaxel and vinorelbine enhancing their antitumor effectiveness.

## Introduction

The development of new blood vessels is needed to provide oxygen and nutrients to the tumor cells and a way to escape from the primary site^1^. Angiogenesis is a main step in tumor growth and antiangiogenic agents are considered an interesting complementary strategy in cancer therapy^2^. Angiogenesis is a multi-step process: endothelial cell activation by growth factors, degradation of capillary wall by proteinases, formation of a branch point in the vessel wall, migration of endothelial cells and proliferation and reorganisation of endothelial cells to form tubules. In tumors, the interaction between endothelial cells and the microenvironment stimulates endothelial cells to proliferate, migrate and form a branching network of tubes^3^. A balance between angiogenic activating and inhibiting factors regulates the evolution of endothelial cells from a quiescent to a proangiogenic stage^2^. Among the principal proangiogenic factors are vascular endothelial growth factor (VEGF) and angiopoietins (ANG)^4,5^. Angiogenesis depends on the coordinated balance in time between the production of VEGF and angiopoietins^6^.

Melatonin, produced in the pineal gland, has antitumor effects in different cancer types, especially hormone-dependent tumors^7–10^. Melatonin oncostatic actions are exerted through different mechanisms, such as: indirect effects through a down-regulation of gonadal estrogens^11^; direct antiestrogenic actions at the tumor cell level^12,13^; induction of apoptosis^14^; antioxidant effects^15^; increase of the anticancer immunity^16^; reduction of telomerase activity^17,18^; inhibition of fatty acid uptake and fat metabolic pathways^19,20^ and inhibition of angiogenesis^21–23^. Concerning to its antiangiogenic effects, in MCF-7 cells melatonin reduces the expression of *VEGF* mRNA and inhibits proliferation, invasion, migration of endothelial cells and tubular network formation induced by VEGF^22–24^. Likewise, melatonin indirectly inhibits angiogenesis through the repression of IGF, EGF and ET-1 (tumor growth factors and enhancers of tumor angiogenesis)^25^ and diminishing the production of ROS, which has an important function in stabilizing hypoxia-inducible factor HIF-α during hypoxia^26^.

Recently, different studies described that melatonin could have enhancing actions on the antineoplastic effects of chemotherapeutic agents^27^. Thus, the disruption of the nocturnal melatonin synthesis generates doxorubicin resistance and the administration of melatonin restores the sensitivity of tumor cells to doxorubicin and produces tumor regression^28^. Melatonin enhances the tunicamycin-induced apoptosis in breast cancer cells^29^ and sensitizes non-small-cell lung cancer cells to gefitinib^30^. In lung and cervical cancer cells melatonin stimulates cisplatin-induced cytotoxicity and apoptosis^31,32^. In addition, in a rat pancreatic tumor cell line, the administration of melatonin with 5-fluorouracil, cisplatin and doxorubicin potentiates chemotherapy-induced cytotoxicity and apoptosis^33^. Recently, our group described in a breast cancer cell line (MCF-7) that melatonin treatment increased the changes provoked by docetaxel on the levels of transcription of some genes (*BAX*, *BAD*, *BCL2*, *TP53*, *CDKN1A*, *MUC1*, *GATA3*, *c-MYC* and *CDH13*) and potentiated the actions on apoptosis^34^. Until now, all the studies about the influence of melatonin on the oncostatic actions induced by chemotherapeutic agents have been realized on tumor cells. Since it has been shown that in human breast cancer melatonin exerts antitumoral actions, some of which act by inhibiting the formation of new blood vessels, and it is also known the beneficial role of melatonin in combination with chemotherapeutics, we wanted in this work to analyze the role of melatonin together with chemotherapy in the various stages of angiogenesis. For this, we used HUVECs treated with chemotherapeutics in the presence or not of melatonin in order to find out if the pineal hormone modifies the possible pro- or anti-angiogenic effects of these drugs. After treating human endothelial cells with docetaxel or vinorelbine, various experiments were carried out to check which levels of angiogenesis (cell viability, migration capacity, capillary formation, expression of factors that favor angiogenesis, etc.) were affected the joint treatment with melatonin, as well as, its effect on the transcription levels of some factors related to angiogenesis. To understand through which mechanisms the indolamine works, in this study, we analyzed some cellular signaling pathways affected by other actions of the hormone. Finally, we studied if melatonin inhibitory effects of angiogenesis were reproducible *in vivo*, using the chorioallantoid membrane assay of chicken embryo.

## Results

### Actions of docetaxel and vinorelbine on endothelial cell proliferation and its modulation by melatonin

**Figure 1:**
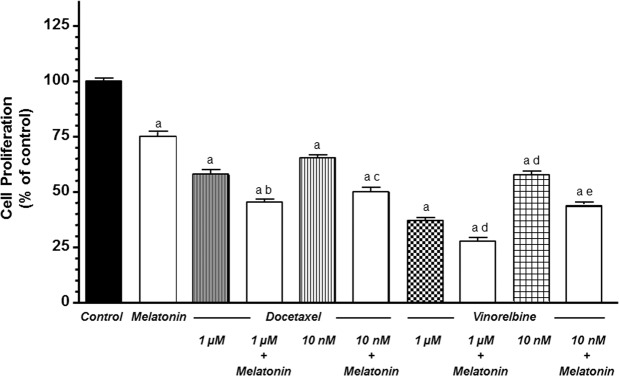
Melatonin increased docetaxel and vinorelbine-induced inhibition of cell proliferation in endothelial cells. Cell proliferation was measured by the MTT method. Data are expressed as the percentage of the control group (mean ± SEM). C control, M melatonin, D docetaxel and V vinorelbine. ^a^p < 0.001 vs control; ^b^p < 0.05 vs D 1 µM; ^c^p < 0.001 vs D 10 nM; ^d^p < 0.05 vs V 1 µM; ^e^p < 0.001 vs V 10 nM.

Firstly, we analysed the effects of docetaxel and vinorelbine and its regulation by melatonin in one of the first phases of angiogenesis such us proliferation of HUVECs. Melatonin at 1 mM alone had an antiproliferative action in HUVECs (Fig. 1). As expected, we observed that both doses (1 µM and 10 nM) of docetaxel and vinorelbine reduced HUVECs proliferation (p < 0.001) in a dose-dependent manner. Moreover, melatonin treatment before chemotherapeutics enhanced the antiproliferative effect exerted by docetaxel and vinorelbine at both concentrations (Fig. 1).

### Actions of docetaxel and vinorelbine on endothelial cell motility and its modulation by melatonin

**Figure 2:**
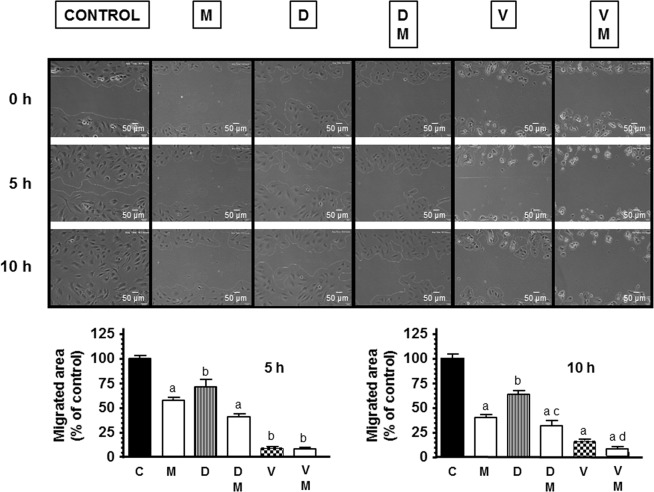
Melatonin 1 mM (M) increased the inhibitory effect on endothelial cell migration induced by docetaxel 10 nM (D) and vinorelbine 10 nM (V). It was analysed through the wound healing assay. Representative photomicrographs of initial and after 5 and 10 h are shown at 10 x magnification and migration distance was measured as described in Material and Methods. Migrated area data shown are expressed as mean ± SEM of four experiments. ^a^p < 0.05 vs C (control); ^b^p < 0.001 vs C; ^c^p < 0.05 vs D; ^d^p < 0.05 vs V.

Since endothelial cell migration is critical for the formation of new bloods during angiogenesis, we studied the consequences of adding melatonin treatment before docetaxel and vinorelbine on the HUVECs migration with the wound-healing assay. In this assay, we used docetaxel and vinorelbine at 10 nM because at 1 µM concentration cells did not migrate. As showed in Fig. 2 docetaxel and vinorelbine treatment for 10 h reduced significantly the distance migrated by HUVECs. Melatonin increased the inhibition on the migratory activity of HUVECs caused by docetaxel and vinorelbine and reduced the rate of wound closure approximately by 50% of cells treated only with docetaxel or vinorelbine.

### Actions of docetaxel and vinorelbine on endothelial cell capillary structure formation and its modulation by melatonin

**Figure 3:**
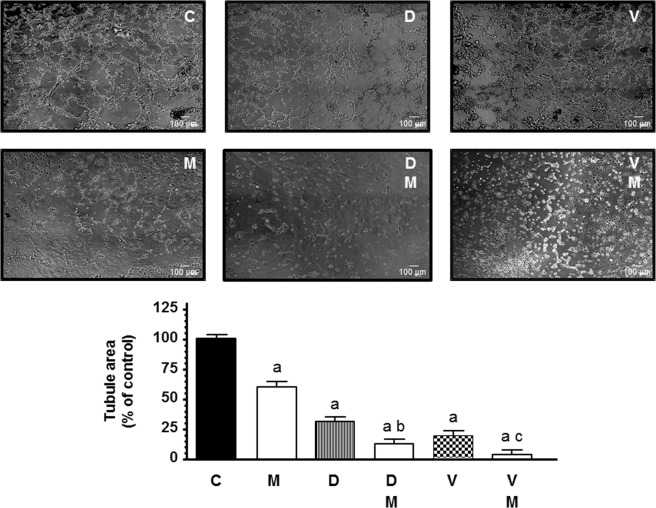
Effects of melatonin 1 mM (M) on docetaxel 10 nM (D) and vinorelbine (V) 10 nM -induced changes on HUVEC capillary structure formation. Tubular structures were photographed and tubule area was measured as described in Material and Methods. Tubule area data are expressed as the percentage of the control group (mean ± SEM). ^a^p < 0.001 vs C; ^b^p < 0.001 vs D; ^c^p < 0.001 vs V.

Following the study of the different phases of angiogenesis, we investigated the capability of HUVECs to form capillary tubes on a matrigel matrix, the effects of docetaxel and vinorelbine in such process and its modulation by melatonin. As in the migration assay, we used docetaxel and vinorelbine at 10 nM. HUVECs seeded on matrigel matrix formed tubular structures after 4 h (Fig. 3). Docetaxel and vinorelbine significantly (p < 0.001) inhibited capillary tube formation. Melatonin 1 mM treatment before docetaxel and vinorelbine reduced the formation of a meshwork of branching capillary-like tubes (Fig. 3) demonstrating a significant (p < 0.001) antivascular effect by enhancing the decrease of tubular structures formed and mesh area produced by both chemotherapeutic agents.

### Actions of docetaxel and vinorelbine as selective estrogen enzyme modulators and its regulation by melatonin

**Figure 4:**
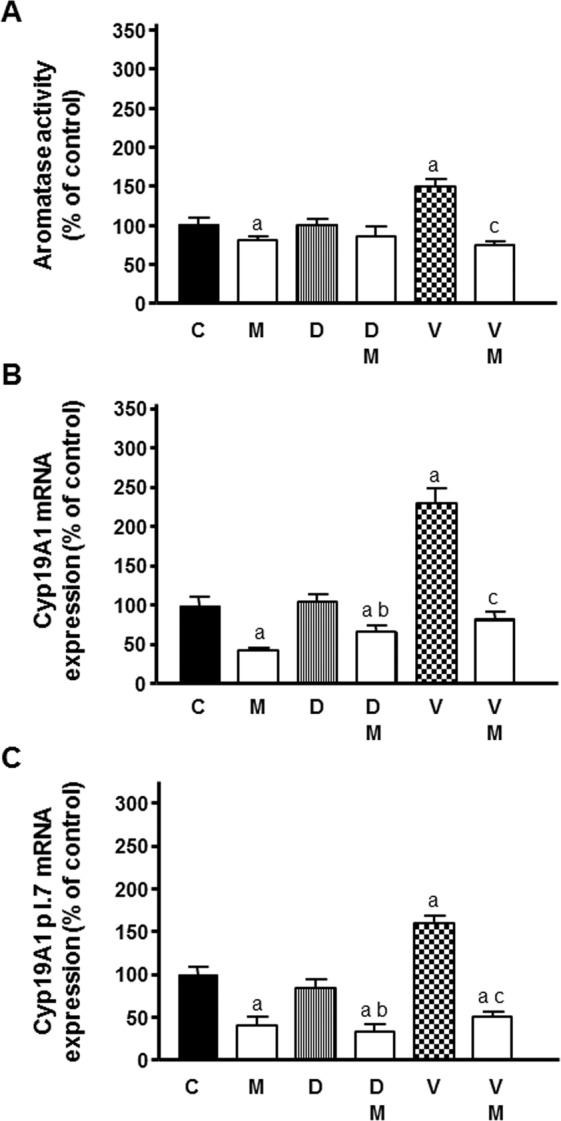
Effects of melatonin 1 mM pretreatment on docetaxel and vinorelbine-induced changes on aromatase activity **(A)** and *Cyp19A1* expression **(B)** and *Cyp19A1 p 1.7* mRNA expression **(C)** in endothelial cells. Data are expressed as the percentage of the control group (mean ± SEM). C control, M melatonin, D docetaxel and V vinorelbine. ^a^p < 0.01 vs C; ^b^p < 0.05 vs D; ^c^p < 0.001 vs V.

Since estrogens play a relevant role in the development and progression of mammary tumors with different effects on endothelium, we studied whether docetaxel and vinorelbine were able to modulate the transcriptional levels and later activity of the enzymes implicated in the estrogen synthesis in HUVECs and its modulation by melatonin. With this aim, we co-cultured HUVECs (lower compartment of the chamber) and MCF-7 (upper compartment of the chamber) cells, due to the fact that malignant cells stimulate the production of estrogens in endothelial cells. Docetaxel did not produced any changes in the aromatase activity of endothelial cells whereas vinorelbine at 1 µM increased aromatase activity. Melatonin pretreatment counteracted this stimulatory effect (Fig. 4A).

With the aim of determining whether the stimulatory effect of vinorelbine on aromatase activity is due to the upregulation of *Cyp19A1* mRNA expression, we perform qRT-PCR with specific primers for *Cyp19A1*. *Cyp19A1* mRNA expression was also significantly stimulated by 1 µM vinorelbine. Treatment with 1 mM melatonin in advance to chemotherapeutics addition reduced the expression of *Cyp19A1* and was able to counteract the stimulatory effect caused by vinorelbine (Fig. 4B).

Since *Cyp19A1 p I.7* is the main aromatase promoter involved in the regulation of *Cyp19A1* expression in breast cancer, we studied by qRT-PCR its expression in endothelial cells. Melatonin decreased *Cyp19A1 p I.7* mRNA expression and counteracted the stimulatory effect induced by vinorelbine (Fig. 4C).

**Figure 5:**
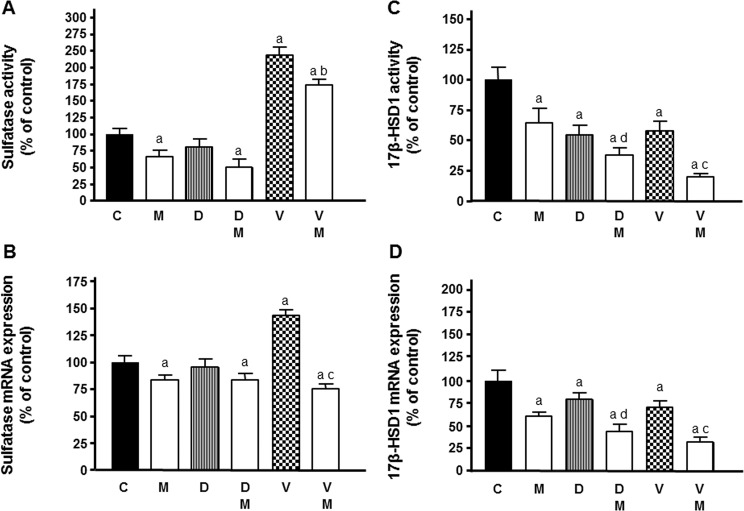
Effects of melatonin pretreatment on docetaxel and vinorelbine-induced changes on sulfatase **(A)** and 17β-HSD1 **(C)** activity and expression **(B**,**D)** in endothelial cells. Data are expressed as the percentage of the control group (mean ± SEM). C control, M melatonin, D docetaxel and V vinorelbine. ^a^p < 0.01 vs C; ^b^p < 0.05 vs V; ^c^p < 0.001 vs V; ^d^p < 0.01 *vs* D.

After that, we analysed whether melatonin could modulate the effects caused by docetaxel and vinorelbine on the activity and expression of sulfatase (STS), the enzyme that synthesizes estrone and 17β-estradiol from sulfated estrogens. Vinorelbine stimulated significantly the activity of this enzyme. Melatonin at 1 mM decreased STS activity in the presence or not of docetaxel and it was able to reduce the stimulatory effect induced by vinorelbine on STS activity (Fig. 5A).

We then wished to determine whether the modulatory effect of vinorelbine on STS activity is due to the regulation of the mRNA expression levels of *STS*. Vinorelbine significantly (p < 0.001) stimulated the expression of *STS* and the treatment with melatonin before chemotherapeutic significantly downregulated the expression of *STS* neutralizing the stimulatory effect induced by vinorelbine (Fig. 5B).

Then, we analysed the effects of docetaxel and vinorelbine on 17β-HSD1 activity and *17β-HSD1* transcription, the enzyme that converts estrone, androstenedione and 5-androstenedione into 17β-estradiol. We also studied whether or not melatonin could regulate these effects. Both docetaxel and vinorelbine reduced the activity of this enzyme. Melatonin also decreased the activity of 17β-HSD1 and significantly enhanced the inhibitory effect exerted by docetaxel and vinorelbine (Fig. 5C).

Docetaxel and vinorelbine downregulated the expression of *17β-HSD1* in HUVECs. Melatonin treatment before chemotherapeutics addition also downregulated the expression of *17β-HSD1* and enhanced the reduction caused by docetaxel and vinorelbine on *17β-HSD1* mRNA expression (Fig. 5D).

### Regulation by melatonin of the docetaxel and vinorelbine-exerted changes on mRNA expression of the main pro-angiogenic factors, the VEGF gene family and angiopoietins

**Figure 6:**
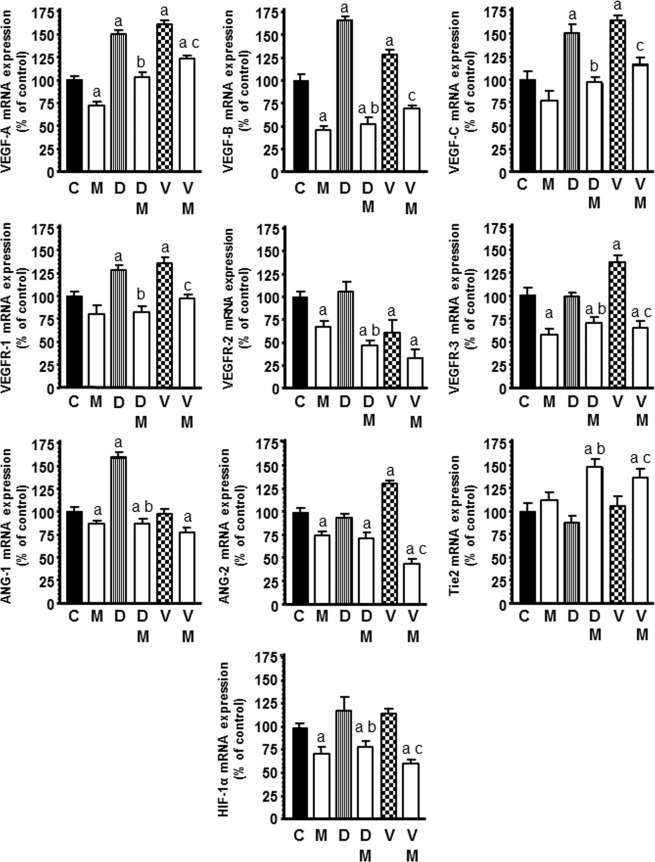
Effects of melatonin on docetaxel and vinorelbine-induced changes on mRNA expression of the main pro-angiogenic factors, *VEGF-A*, *VEGF-B*, *VEGF-C*, *VEGFR1*, *VEGFR2*, *VEGFR3*, *ANG-1*, *ANG-2*, *Tie 2* and *HIF-1α*. Data are expressed as the percentage of the control group (mean ± SEM). C control, M melatonin, D docetaxel and V vinorelbine. ^a^p < 0.001 vs C; ^b^p < 0.001 vs D; ^c^p < 0.001 vs V.

Docetaxel at 1 µM induced a significant increase in the expression of *VEGF-A*, *VEGFB*, *VEGF-C*, *VEGFR-1* and *ANG-1* and the treatment with melatonin before chemotherapeutic caused a significant decrease of the expression of these angiogenic factors neutralizing the stimulatory effect induced by docetaxel (Fig. 6). *VEGF-A*, *VEGF-B*, *VEGF-C*, *VEGFR-1*, *VEGFR-3* and *ANG-2* mRNA expression was also upregulated by vinorelbine and melatonin pretreatment counteracted this stimulatory effect induced by vinorelbine (Fig. 6). Vinorelbine also downregulated *VEGFR-2* and melatonin potentiated this inhibitory effect. Melatonin in combination with docetaxel and vinorelbine increased *Tie2* mRNA expression, the angiopoietins cognate receptor (Fig. 6). Docetaxel and vinorelbine did not modify the expression of *HIF-1α*, however, melatonin decreased its expression with or without both chemotherapeutics.

### Actions of docetaxel and vinorelbine on the expression of genes associated with angiogenesis and its modulation by melatonin

**Figure 7:**
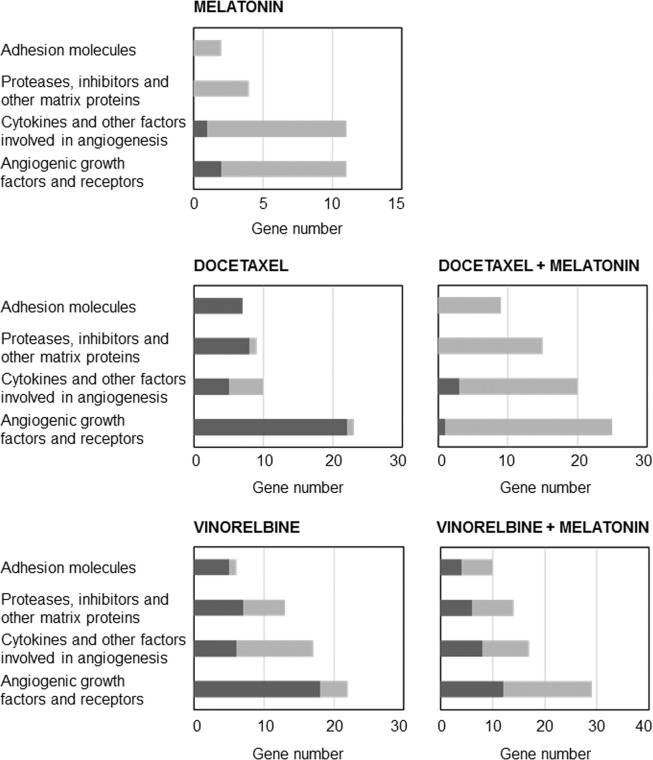
Genes induced or repressed at least two-fold in endothelial cells treated either with docetaxel 1 µM or vinorelbine 1 µM and/or melatonin 1 mM for 4 h. Pathway-focused gene expression profiling was performed using a 96-well human breast cancer Human Angiogenesis RT^2^ Profiler PCR Array. Black columns indicate the percentage of upregulated genes and gray columns indicate the percentage of downregulated genes in each category.

**Figure 8:**
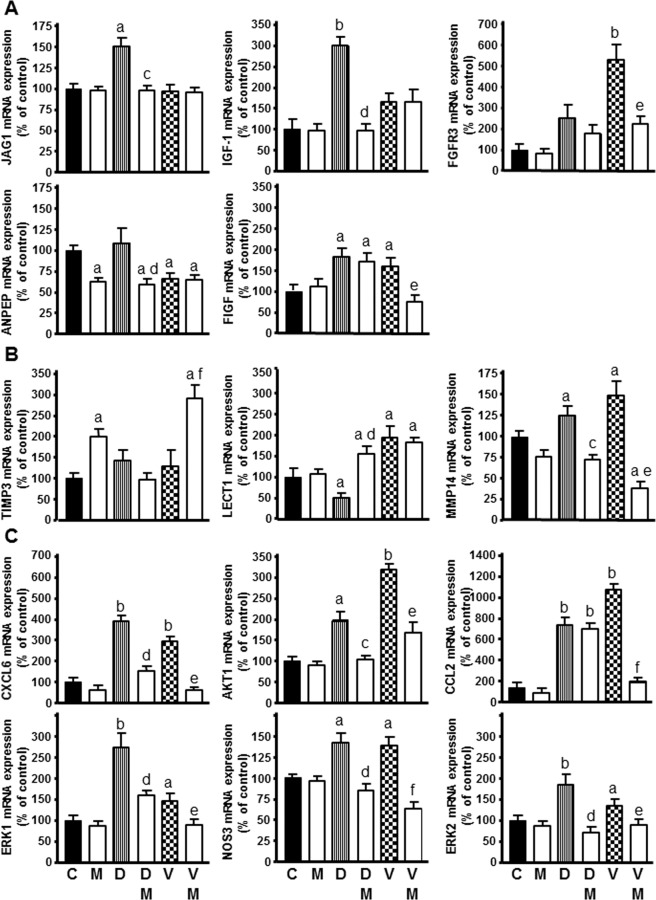
Effect of docetaxel 1 µM and vinorelbine 1 µM in the presence or not of melatonin 1 mM on the genes selected for analysis by specific RT-qPCR: **(A)** *JAG1*, *IGF-1*, *FGFR3*, *ANPEP* and *FIGF* as angiogenic growth factors, **(B)** *TIMP3*, *MMP14* and *LECT1* as extracellular matrix molecules, **(C)** *CXCL6*, *NOS3*, *CCL2*, *ERK1*, *ERK2* and *AKT1* as cytokines and other angiogenic factors. Data are expressed as the percentage of the control group (mean ± SEM). C control, M melatonin, D docetaxel and V vinorelbine. ^a^p < 0.01 vs C; ^b^p < 0.001 vs C; ^c^p < 0.05 vs D; ^d^p < 0.001 vs D; ^e^p < 0.05 vs V; ^f^p < 0.001 vs V.

Since chemotherapeutics agents-induced modifications of gene expression associated with the angiogenic process are not well known, we employed the Human Angiogenesis RT^2^ Profiler PCR Array, which analyses the expression of 84 genes implicated in angiogenesis. Figure 7 shows the number of genes induced or repressed by the different treatments. Docetaxel enhanced the expression of 42 genes and decreased the expression of 7 genes. Vinorelbine induced the upregulation of 36 and the downregulation of 22 genes. Melatonin alone downregulated 25 and upregulated 3 genes. Melatonin with docetaxel downregulated 65 and upregulated 4 genes. In combination with vinorelbine, melatonin downregulated 40 and upregulated 30 genes (Supplementary File with the expression changes of the 84 genes studied). Then, we selected for analysis by specific RT-qPCR those that had been modified under two or more treatments and we excluded those that we had analysed in the previous experiments (*VEGF-A*, *VEGF-B*, *VEGF-C*, *VEGFR-1*, *VEGFR-2*, *VEGFR-3*, *HIF-1α*, *ANG-1*, *ANG-2*, *Tie2*) and those whose modulation by melatonin has been previously reported. The effects on different angiogenic factors modified by docetaxel and vinorelbine with or without melatonin are shown in Fig. 8, grouped in three categories.

The first category compiles the angiogenic growth factors. Docetaxel enhanced the expression of *FIGF*, *IGF-1* and *JAG1*. Vinorelbine upregulated *FGFR3* and *FIGF* expression and downregulated *ANPEP*. Melatonin neutralized the stimulatory effect of docetaxel on the expression of *JAG1*, *ANPEP* and *IGF-1* and also neutralized the stimulation of vinorelbine on the expression of *FIGF* and *FGFR3* (Fig. 8A).

Secondly, extracellular matrix molecules related to angiogenesis. Docetaxel decreased *LECT1* expression and increased *MMP14* expression. Vinorelbine upregulated *LECT1* and *MMP14*. Melatonin was able to counteract the actions of docetaxel and vinorelbine on the expression of *MMP14*. Likewise, melatonin neutralized the decrease induced by docetaxel by increasing *LECT1* expression and increased *TIMP3* expression in combination with vinorelbine (Fig. 8B).

Cytokines and other angiogenic factors belong to the third group. Both docetaxel and vinorelbine enhanced the expression of *CXCL6*, *NOS3*, *CCL2*, *ERK1*, *ERK2* and *AKT1*. Melatonin neutralized the stimulatory effect of both docetaxel and vinorelbine on the expression of *CXCL6*, *NOS3*, *ERK1*, *ERK2* and *AKT1*. Melatonin also counteracted the stimulatory action of vinorelbine on the expression of *CCL2* (Fig. 8C).

### Actions of docetaxel and vinorelbine on AKT and ERK signaling and its modulation by melatonin

**Figure 9:**
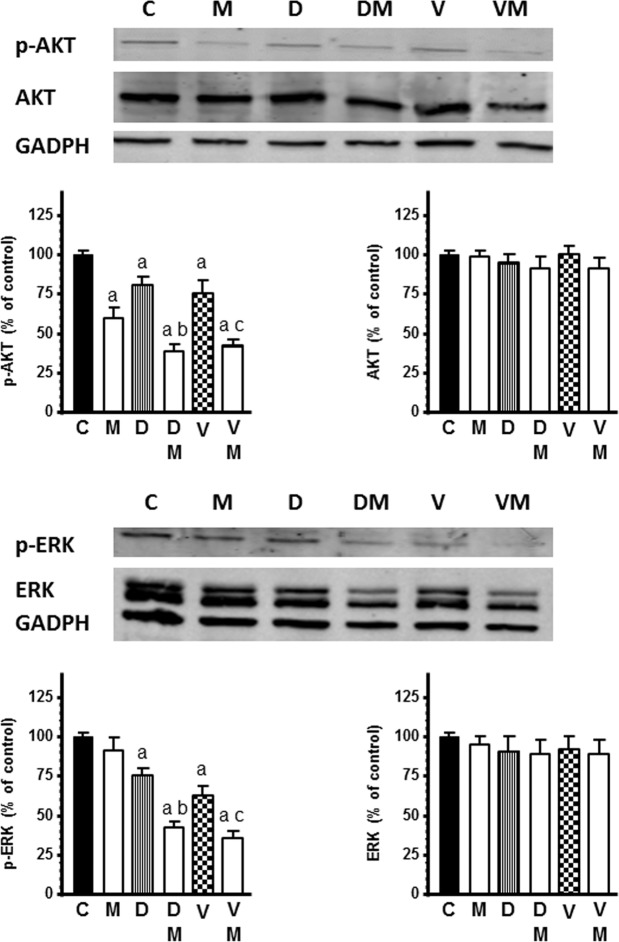
Effects of docetaxel 1 µM and vinorelbine 1 µM in the presence or not of melatonin 1 mM on AKT and ERK signaling pathways. Western blot representative images and analyses showing protein levels for AKT, ERK, p-AKT, p-ERK and GADPH as a control. Data are expressed as the percentage of the control group (mean ± SEM). C control, M melatonin, D docetaxel and V vinorelbine. ^a^p < 0.01 *vs* C; ^b^p < 0.001 *vs* D; ^c^p < 0.01 *vs* V.

Total AKT and ERK protein expression was not affected by docetaxel and vinorelbine, but the contents of p-AKT and p-ERK were decreased in the presence of both chemotherapeutics. On the other hand, melatonin 1 mM potentiated the inhibitory effect of docetaxel and vinorelbine on the phosphorylation levels of AKT and ERK (Fig. 9).

### Actions of docetaxel and vinorelbine on vascular permeability and its modulation by melatonin

**Figure 10:**
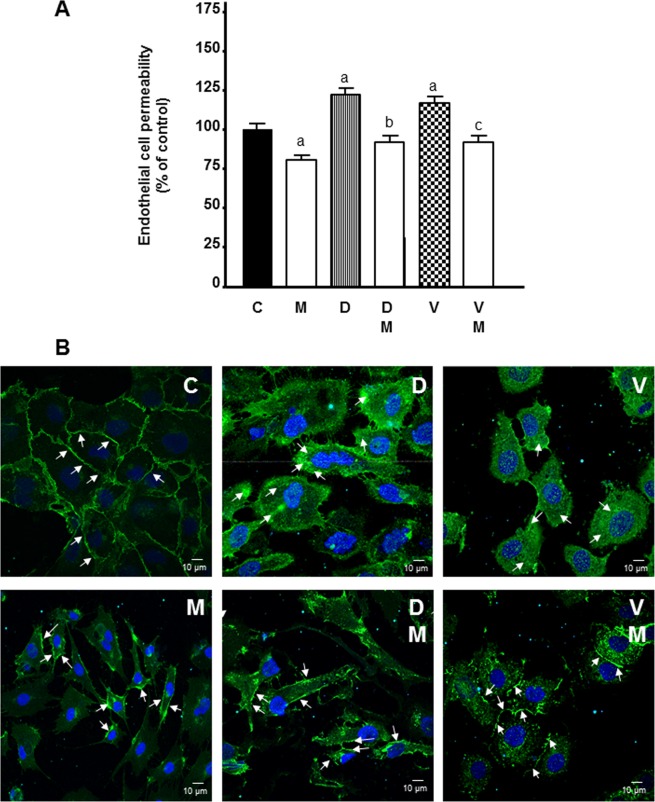
Effects of melatonin on docetaxel 1 µM and vinorelbine 1 µM -induced changes on endothelial cell permeability (A) and VE-cadherin internalization (B). Arrows show VE-cadherin staining in cell to cell contact, in control and melatonin treated groups, and intracellular accumulation, in docetaxel and vinorelbine treated groups. Data are expressed as the percentage of the control group (mean ± SEM). C control, M melatonin, D docetaxel and V vinorelbine. ^a^p < 0.05 *vs* C; ^b^p < 0.01 *vs* D; ^c^p < 0.01 *vs* V.

With the objective of analysing if melatonin in combination with docetaxel or vinorelbine could affect vascular normalization, we addressed the effects of docetaxel and vinorelbine on endothelial cell permeability and its modulation by melatonin. Docetaxel and vinorelbine enhanced permeability of the HUVECs and melatonin was able to neutralize this effect (Fig. 10A).

VE-cadherin is one of the essential junctional molecule controlling the endothelial monolayer permeability, and since we have found that melatonin decreased cell permeability, we considered studying whether melatonin modified the distribution of VE-cadherin by immunostaining. In control endothelial cells, cell surfaces showed VE-cadherin staining. In contrast, docetaxel and vinorelbine disrupted this pattern and induced the internalization of the VE-cadherin and its disappearance from cell-to-cell contact. Melatonin reduced the internalization of the VE-cadherin induced by docetaxel and vinorelbine and enhanced VE-cadherin accumulation at cell surface (Fig. 10B).

### *In vivo* actions of docetaxel and vinorelbine on the formation of new blood vessels and its modulation by melatonin

**Figure 11:**
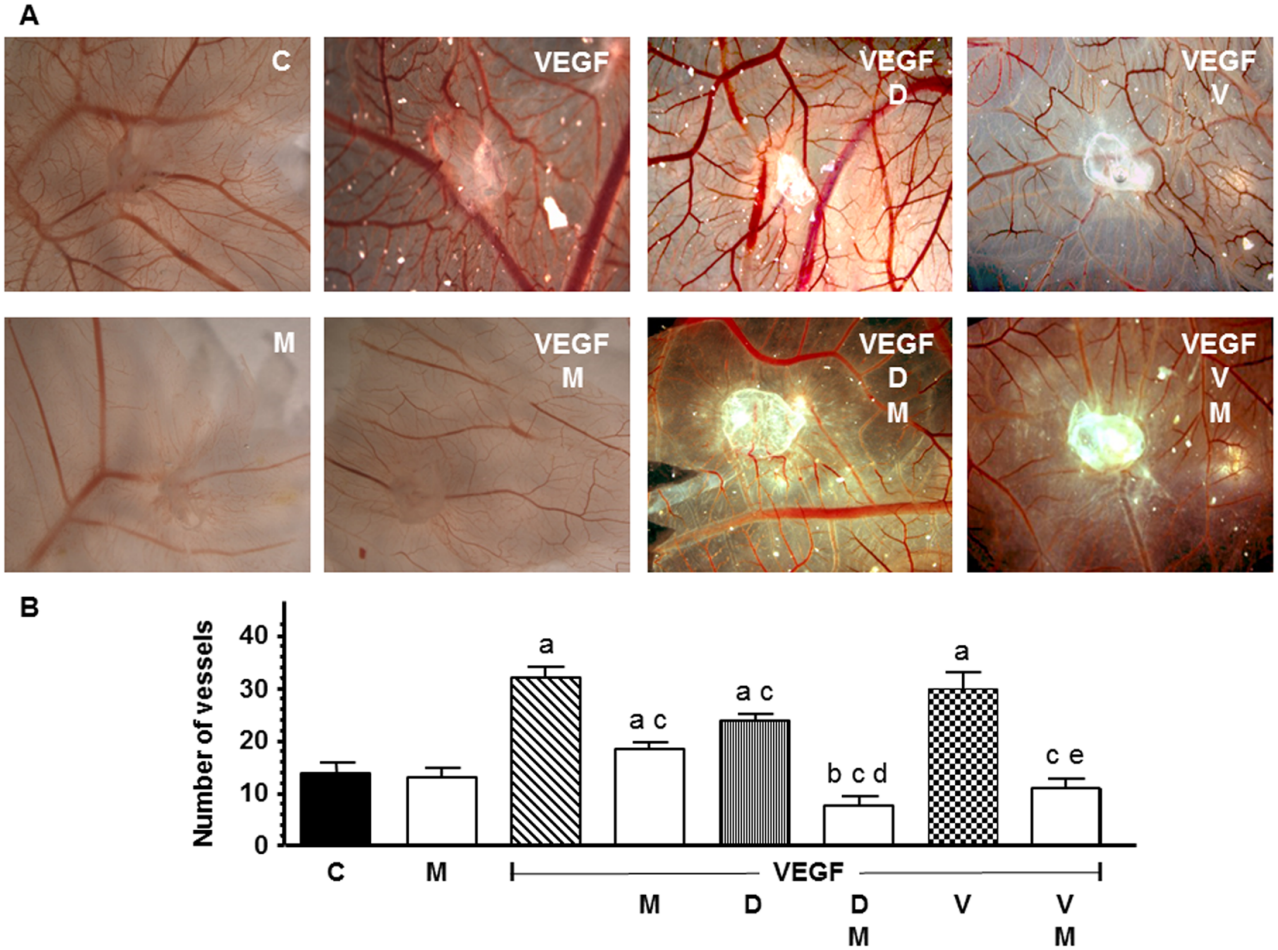
*In vivo* effect of melatonin on the docetaxel or vinorelbine-induced changes on the VEGF-stimulated angiogenesis in the chorioallantoic membrane assay. **(A)** Representative images of CAM. **(B)** Vessel number data shown are expressed as mean ± SEM. C control, M melatonin, VEGF vascular endothelial growth factor, D docetaxel and V vinorelbine. ^a^p < 0.001 vs C; ^b^p < 0.01 vs C; ^c^p < 0.001 vs VEGF; ^d^p < 0.001 vs VEGF D; ^e^p < 0.001 vs VEGF V.

We assessed the formation of new vessels in the chick CAM assay. VEGF alone induced numerous blood vessels (Fig. 11A). The number of new formed vessels induced by VEGF was reduced by docetaxel treatment. In addition, melatonin treatment potentiated significantly (p < 0.001) the decrease of the number of vessel branch points produced by docetaxel and also decreased number of vessels in combination with vinorelbine (Fig. 11B).

## Discussion

The formation of new vessels is essential in tumor ontogenesis and evolution. Inhibition of tumor angiogenesis has converted to an anticancer treatment in agreement with chemotherapy, radiotherapy or surgery^35^. Chemotherapeutic agents are sometimes administered with other drugs targeting different pathways that cause synergism and higher effectiveness of treatment. However, chemotherapeutic agents are frequently accompanied by deleterious side effects and development of drug resistance. Chemotherapeutic agents are classified according to their modes of action, as they act on different cellular targets affecting divers pathways which could cause cell cycle arrest and/or the induction of apoptosis. One of the most successful targeted therapy involves the microtubule inhibitors, such as docetaxel, microtubule polymerizing agent, or vinorelbine, microtubule depolymerizing agent. They inhibit microtubule functions, which are required during cancer cell division^36,37^.

Combined with classical chemotherapeutic agents, melatonin enhances the efficacy of these drugs in breast cancer patients via a variety of mechanisms, including the capability of impairing angiogenesis^21,22,27,34^. Thus, in the present study, we analysed the effects of docetaxel and vinorelbine at different stages of angiogenesis, comparing the efficacy of the drugs alone or added together with the pineal hormone.

Here in, we tried to demonstrate that indeed melatonin both stimulates the antiangiogenic actions and abrogates any proangiogenic responses to docetaxel or vinorelbine. We have designed different experiments to assess different phases of the angiogenic response. First, we examined the cell division ratio of HUVECs. Docetaxel and vinorelbine, as expected, reduced endothelial cell proliferation. As we have already described in other works, melatonin at 1 mM inhibited the proliferation of endothelial cells^23^. Besides, pretreatment with the pineal hormone further stimulates the inhibition of cell proliferation exerted by docetaxel and vinorelbine. This finding matches with previous results of our group: melatonin enhances apoptosis, further represses cell proliferation and is able to alter the modifications in gene expression triggered by docetaxel in the estrogen-responsive MCF-7 cell line^34^. The next stage of angiogenesis we tested was cellular migration. Docetaxel or vinorelbine reduced the migration capability of endothelial cells, in agreement with previously reports, describing that both vinorelbine and docetaxel inhibit endothelial cell motility and angiogenesis. In the case of docetaxel, by inhibiting the dynamic instability of microtubules that possibly interact with G-proteins associated with focal adhesions and through the VE-cadherin mediated integrin β1/FAK/ROCK signalling pathway^38–40^.

In our study, the association of melatonin pretreatment with these chemotherapeutics agents further reduced by 50% the distance migrated by HUVECs in contrast to those groups treated only with docetaxel or vinorelbine. Once again melatonin enhanced docetaxel and vinorelbine effects, in this case on the migration of endothelial cells. Our results are in concordance with previous works describing that the inhibitory actions on angiogenesis exerted by melatonin on endothelial cells might take account by inhibition of cell migration^23^.

Since the development of tubes by endothelial cells is another important step in neo-angiogenesis, we studied the effect of docetaxel and vinorelbine, in the presence or not of melatonin, in an endothelial cell-vessel formation on matrigel matrix. Docetaxel and vinorelbine inhibit the three-dimensional configuration of our cell model into structures resembling capillary tubes^41,42^. In this study, we found that both docetaxel and vinorelbine decreased also the tubular network formation and melatonin disrupted tube assembly and markedly reduced the tubule area observed in response to both chemotherapeutics agents. In agreement, a previously published work indicates that the pineal hormone disrupts tube assembly, thwarts the arrangement of endothelial cells in a capillary network and dismantles those capillary networks assembled before treatment^23^.

It has been described how estradiol can accelerate the endothelial cell proliferation both *in vitro* and *in vivo*, promotes endothelial cell adhesion to a variety of matrix proteins and enhances cellular migration, ultimately leading to angiogenesis^43^. ERα transactivation seems to be one of the main mechanisms underlying vessel formation. In breast cancer, the action of estrogens, the expression of ER by endothelial cells correlates with angiogenic effects and the ability of the tumor to invade^43^. For this reason, we studied the effect of docetaxel and vinorelbine on the transcription and activity of some enzymes (17β-HSD1, aromatase and estrogen sulfatase) participating in estrogen production in HUVECs. In our study, the greatest effects were obtained with vinorelbine, which induced an increase in aromatase and sulfatase transcription and enzymatic activity, whereas melatonin pretreatment reverted this response. Vinorelbine increased also *Cyp19A1 p I.7* expression, the main promoter driving aromatase transcription in endothelial cells in mammary tumors. Melatonin significantly downregulated the *Cyp19A1 p I.7* and also counteracted the stimulatory effect induced by vinorelbine. Vinorelbine and docetaxel downregulated 17β-HSD1 transcription and inhibited its enzymatic activity. In this case, melatonin pretreatment markedly reinforced this effect. Of note, melatonin neutralized the negative effect of vinorelbine, decreasing aromatase and sulfatase activity and expression. At this moment, the effect of docetaxel and vinorelbine on the metabolism of estrogens in endothelial cells is not completely well established. It has been described that docetaxel decreases Cyp19A1 mRNA levels in mammary tumors, thus indicating that the anticancer actions of chemotherapy might account, at least partially, on its ability to suppress estrogens synthesis^44^.

VEGF is probably the most active signal protein directly stimulating the different stages of angiogenesis on endothelial cells^45^. Moreover, the ANG/Tie2 signalling complex is, together with VEGF, the other main molecular inductor of tumor angiogenesis^5,46,47^. Angiopoietins (ANG-1 and ANG-2) and VEGF act in a complementary and organized manner. Both angiopoietins can bind to the Tie2 receptor (belonging to the family of tyrosine kinases). ANG-1 promotes vascular maturation and stabilization. ANG-2 is an antagonist for the Tie2 receptor and causes vascular regression if VEGF is absent, whereas it stimulates angiogenesis when VEGF is present^48,49^. Docetaxel induced an increase in *VEGF-A*, *VEGFB*, *VEGF-C*, *VEGFR-1* and *ANG-1* transcription, whereas melatonin significantly decreased these angiogenic factors neutralizing the stimulatory effect induced by docetaxel. At the same time, melatonin with docetaxel or vinorelbine upregulated *Tie2* transcription in HUVECs. We know that the angiopoietins-Tie2 system has an autoregulation feedback system that modulates the overall activity of the Tie2 system so that ANG-1, but not ANG-2 down-regulates Tie2 mRNA expression in endothelial cells^50^. The downregulation induced by melatonin on *ANG-1* expression could explain the stimulatory effect of melatonin on *Tie2* expression. Vinorelbine upregulated *VEGF-A*, *VEGF-B*, *VEGF-C*, *VEGFR-1*, *VEGFR-3* and *ANG-2* transcription and the pineal hormone abolished this stimulatory effect induced by vinorelbine. Importantly, the induction of docetaxel and vinorelbine on *VEGF-A*, *VEGF-B*, *VEGF-C*, *VEGFR-1*, *VEGFR-3*, *ANG-1* and *ANG-2* expression should be regarded as a non-desirable side effect since they are increasing angiogenesis. To contrary, melatonin abolished this adverse effect and reduced their expression. In breast cancer, stimulatory effects in VEGF and ANG-1 after chemoendocrine therapy, such as doxorubicin, docetaxel, tamoxifen, exemestane or letrozol, have been described with low concentrations^51^. It has been already found that melatonin diminishes the levels of VEGF in several breast cancer cell lines and simultaneously triggers a decrease in the level of angiopoietins with a reduction of VEGF when endothelial and human breast cancer cells are cocultured^22,23^.

The same antiangiogenic properties of melatonin, mediated through a decline in the levels of VEGF has been described in several kind of tumors, such as neuroblastoma^52^, breast cancer^22,23^, ovarian^53^, gastric^54^, pancreatic^55^, prostate^56^ or hepatocarcinoma^57^. Additionally, the pineal hormone concomitantly administered with cyclophosphamide reduced *VEGF-A* transcription and tumor growth, both in salivary glands and Walker 256 carcinoma^58^.

We also addressed the transcription rate of 84 genes involved in angiogenesis in endothelial cells treated with docetaxel or vinorelbine in the presence or not of melatonin, in order to study whether chemotherapeutic agents modified some of these angiogenic factors and whether melatonin was able to counteract or to potentiate these effects. We further analyzed the expression of *JAG1*, *ANPEP*, *FGFR3*, *IGF-1* and *FIGF* (growth factors promoting angiogenesis), *TIMP3*, *MMP14* and *LECT1* as extracellular matrix proteins and finally, a group of cytokines and other factors stimulators of angiogenesis, such as *NOS3*, *CXCL6*, *ERK1*, *ERK2, CCL2* and *AKT1*. To study by specific RT-PCR we chose these genes, because they were altered in several experimental groups and, to date, it is not known whether melatonin is able to modulate them or not.

Concerning the regulatory actions on angiogenic factors, docetaxel upregulated *FIGF*, *IGF-1* and *JAG1* transcription. Vinorelbine upregulated *FGFR3* and *FIGF* expression and downregulated *ANPEP*. *JAG1* is involved in cancer development at different levels, since it promotes tumor angiogenesis, stimulates malignant cell growth and triggers metastasis^59^. Transcription of *JAG1* is elevated in mammary tumors and correlates with bad prognosis and low expectations of overall breast cancer survival^59,60^. In non-small cell lung cancer, in tumor-associated endothelial cells, high *ANPEP* levels are in association with angiogenesis and poor prognosis, as determined by microvessel density and/or *VEGF* expression^61^. *IGF-I* promotes tumor cell proliferation, malignant transformation, development of new vessels and tumor development^62^. *FGFR3* high levels directly correlate with poor prognosis in mammary tumors^63^. Additionally, recent reports suggest that *FGFR3* may participate in a molecular pathway leading to breast cancer development. This fact indicates that the FGFR3 protein might be a target to be considered in *FGFR3*-related breast cancer^63^. *FIGF* is related with tumor cell migration in colorectal cancer metastasis^64^. Here we describe that melatonin abolished the induction of transcription of *JAG1*, *ANPEP* and *IGF-1* mediated by docetaxel, and abolished the induction triggered by vinorelbine on the expression of *FIGF* and *FGFR3*. Chemotherapeutic agents upregulate angiogenic growth factors and melatonin, once again, neutralizes these undesirable effects.

Concerning the regulation of extracellular matrix proteins implicated in the angiogenic process, docetaxel decreased *LECT1* expression and increased *MMP14* expression. Vinorelbine upregulated *LECT1* and *MMP14. MMP14* is a metalloproteinase playing a crucial role promoting invasion and metastasis. This metalloproteinase cleaves proteins of the extracellular matrix and also of the basement membrane. As a result, growth factors, MMP-2 and MMP-13, are activated and cells can escape, migrating from the original tumor^65^. *LECT1* inhibits the proliferation and tube morphogenesis of vascular endothelial cells *in vitro* as well as angiogenesis in the chick chorioallantoic membrane and impairs the VEGF-A-stimulated motility of endothelial cells by destabilizing lamellipodial extensions^66^. Our findings suggest that melatonin can abolish the stimulation exerted by docetaxel and vinorelbine on *MMP14* expression and reverted the decrease of *LECT1* expression consequence of docetaxel treatment. Melatonin, alone or in combination with vinorelbine, also increased *TIMP3* expression, an inhibitor of angiogenesis that limits vessel density close to tumors and reduces tumor growth^67^. Recently, it has been described that melatonin reduces Dalton’s lymphoma-induced angiogenesis by increasing *TIMP3* expression^68^.

With respect to other factors implicated in angiogenesis, we found that both docetaxel and vinorelbine increased *AKT1, ERK1*, *ERK2*, *CXCL6*, *NOS3* and *CCL2* transcription. *CXCL6* stimulates breast cancer development triggering the phosphorylation of ERK1 and ERK2, thus inducing migration and growth of endothelial cells^69^. *CCL2* turns human breast epithelial cells into a more aggressive and invasive phenotype^70^. *NOS3* transcription has been correlated with malignancy in human mammary cancer^71^. The protein kinases AKT1, ERK1 and ERK2 participate in multitude of signaling transduction intracellular pathways. In the case of endothelial cells, these protein kinases activate a great number of proteins, which in turn stimulate cell division, cell survival, accelerate cell metabolism and induce vascular remodeling^72^. Melatonin annulled the activation in response to docetaxel and vinorelbine on *CXCL6*, *NOS3*, *ERK1/2* and *AKT1* transcription and abolished the stimulation of vinorelbine on *CCL2* expression. In a cell-line model of hepatocellular carcinoma, the pineal hormone downregulated *CXCL6* at high concentrations^73^. Recent reports link the reversion of drug resistance (in cancer chemotherapy treatments) by melatonin with its ability to inactive AKT and MAPK intracellular signal transduction pathways^74^. In our model, melatonin inhibited the transcription of *AKT1* and *ERK ½*. Both vinorelbine and docetaxel prevent the activation of p-AKT and p-ERK and the pineal hormone enhanced this effect. In summary, our results point to melatonin and a molecule able to inhibit HUVEC cells proliferation, with antiangiogenic properties, effects likely mediated through AKT and ERK intracellular pathways, since it is well known the implication of these two kinases in different stages of angiogenesis: vascular endothelial cell growth, survival, proliferation and migration, ultimately leading to angiogenesis.

Recently, our group described that melatonin abolished the induction of HUVECs permeability in response to radiation. Cell permeability is crucial for vascular function. Therefore, we studied if docetaxel and vinorelbine could also influence permeability and the effects of melatonin in this process. Indeed, both chemotherapeutics induced endothelial cell permeability; interestingly, the pineal hormone impaired their stimulatory effect. It is known that docetaxel^75^ and vinorelbine^76^ increases in permeability are accompanied by changes in vascular endothelial (VE)-cadherin. VE-cadherin belongs to the family of junction proteins and, in endothelial cells, participates in the regulation of vascular permeability^77^. VE_cadherin levels increase in cell to cell contact. Docetaxel and vinorelbine treatments result in intracellular accumulation of this adhesion protein. Melatonin reverted the action of chemotherapeutics decreasing the amount of intracellular VE-cadherin. Phosphorylation of VE-cadherin results in internalization of this adhesion molecule, and, as a consequence, the permeability of endothelial cells is increased^77^. This event is stimulated by VEGF, a permeability-increasing factor^78^. Once more, the inhibitory effect of the pineal hormone on VEGF levels by melatonin might be the explanation for the loss of cell permeability triggered by docetaxel and vinorelbine. Besides, the PI3K/AKT and MAPK/ERK signalling pathways are known to play a role in the increase of permeability exerted by VEGF in endothelial cells^79^. Thus, melatonin could regulate the cell permeability by decreasing the internalization of VE-cadherin, likely through a decrease of VEGF and blocking the phosphorylation of both ERK and AKT.

Additionally, we studied melatonin antiangiogenic activity in the CAM assay as a model of neovessel formation. According to the *in vitro* studies both docetaxel and vinorelbine diminished the vascular area and the pineal hormone intensified this effect. To our knowledge our results are the first ones demonstrating a inhibitory effect of the pineal hormone on angiogenesis when concomitantly administered with some chemotherapeutics in an *in vivo* model.

Our results suggest that melatonin should be considered by its potential to improve the effects of conventional chemotherapeutic agents in breast cancer treatment. Most of our study has been performed *in vitro*. Further *in vivo* studies and clinical trials are necessary to clarify the efficacy of the association of melatonin with chemotherapeutics, like docetaxel or vinorelbine.

In summary, melatonin showed inhibitory effects on angiogenesis in human endothelial cells in the presence of two chemotherapeutics, docetaxel and vinorelbine, by potentiating antiangiogenic actions and neutralizing proangiogenic actions induced by the chemotherapeutics agents. Chemotherapeutic agents inhibit tumor growth through different actions that reduce tumor angiogenesis. However, at the same time, they have actions that are proangiogenic, for example, vinorelbine increased aromatase activity, Cyp19A1 mRNA expression, *Cyp19A1 p I.7* mRNA expression, sulfatase activity, *STS* mRNA expression, *VEGF-A*, *VEGF-B*, *VEGF-C*, *VEGFR-1*, *VEGFR-3* mRNA expression, *ANG-2* mRNA expression, *FGFR3*, *FIGF*, *MMP14*, *CXCL6*, *CCL2*, *AKT1* and *NOS3* mRNA expression. Docetaxel increased *VEGF-A*, *VEGFB*, *VEGF-C* and *VEGFR-1* mRNA expression, increased *ANG-1* mRNA expression, *JAG1*, *FIGF*, *IGF-1*, *MMP14*, *CXCL6*, *AKT1*, *NOS3* mRNA expression and decreased *LECT1* transcriptional activity. Eventually, all these actions stimulate tumor growth. Melatonin treatment prevented all these deleterious effects of chemotherapeutic agents. Antiangiogenic effects of melatonin are likely accomplished through AKT and ERK inhibition since the pineal hormone diminished the phosphorylation levels induced by chemotherapeutics. Moreover, melatonin interferes with VEGF reducing angiogenesis by diminishing cell permeability and modulating the internalization of VE-cadherin. Although it is known that melatonin reverts chemoresistance to chemotherapeutic agents in breast cancer treatment, the most valuable part of this work have been to find that docetaxel and vinorelbine have some non-desirable side effects that interfere with its effectiveness against cancer progression. Importantly, the pineal hormone neutralizes the negative actions of chemotherapeutic agents enhancing their antitumor action.

## Materials and Methods

### Cells and culture conditions

Human umbilical vein endothelial cells (HUVECs) were purchased from the American Tissue Culture Collection (Rockville, MD, USA). Based in previous works^21,22^, we cultured HUVECs as monolayer cultures in 58,1 cm^2^ culture plates in Vascular Cell Basal Medium (VCBM) (ATCC, Rockville, MD, USA) enriched with endothelial cell growth kit-BBE (ATCC, Rockville, MD, USA) which contains 2% fetal bovine serum (FBS), 0.2% Bovine Brain Extract, 5 ng/ml rhEGF, 10 mM Lglutamine, 0.75 units/ml heparin sulfate, 1 µg/ml hydrocortisone hemisuccinate, 50 µg/ml ascorbic acid, penicillin (20 units/ml) and streptomycin (20 µg/ml) (Sigma-Aldrich, Madrid, Spain) at 37 °C in a humid atmosphere with 5% CO_2_.

MCF-7 human breast cancer cells were also obtained from the American Tissue Culture Collection (Rockville, MD, USA). Based in previous works^23^, MCF-7 cells were cultured in 75 cm^2^ plastic culture flasks in Dulbecco’s Modified Eagle’s Medium (DMEM) (Sigma-Aldrich, Madrid, Spain) with 10% fetal bovine serum (FBS) (PAA Laboratories, Pasching, Austria), penicillin (20 units/ml) and streptomycin (20 μg/ml) (Sigma-Aldrich, Madrid, Spain) at 37 °C in a humid atmosphere with 5% CO_2_.

Since it is known that the presence of malignant epithelial cells in the culture enhances estrogen formation in endothelial cells we employed co-cultures of HUVECs and MCF7. As in previous works^22^, cells were co-cultured using Falcon 6-multiwell plates and Falcon cell culture inserts. HUVECs, which have been treated for a week with melatonin 1 mM or its diluent, were seeded (50 × 10^4^ cells/well) on the bottom wells in VCBM enriched with 2% FBS and incubated overnight. Simultaneously, MCF-7 cells (40 × 10^4^ cells), which have been treated for a week with melatonin 1 mM or its diluent, were cultured on the permeable membrane (0.45 µm) of the tissue-culture inserts in DMEM supplemented with 10% FBS. Both types of cells were cultured separately for 24 h to facilitate adhesion. Then, MCF-7 seeded inserts were moved over HUVECs cell cultures in the 6-well plates in fresh VCBM supplemented with 2% FBS to create the hanging co-culture setup. After 24 h, media were changed to VCBM with 2% FBS or serum free, according to the experiment, containing docetaxel 1 µM or vinorelbine 1 µM or its diluent (ethanol at a final concentration lower than 0.001%) for 4 h to measure specific mRNA gene expression or for 0.5 or 24 h to measure the different enzymatic activities.

### Measurement of cellular proliferation

Because the reduction of tetrazolium salts is extensively accepted as a confident way to study cell viability and proliferation, we used the MTT [3(4,5dimethylthiazol-2-yl)-2,5-diphenyl tetrazolium bromide] assay^80^. MTT was obtained from Molecular Probes Inc. (Eugene, OR, USA). As in previous studies^22^, cells were cultured for one week in VCBM with 2% FBS and melatonin (1 mM) (Sigma-Aldrich, Madrid, Spain) or its diluent (ethanol at a final concentration lower than 0.001%). Then, both melatonin pretreated and control cells were cultured into 96-multiwell plates at a density of 8 × 10^3^ cells/well, and incubated at 37 °C for 24 h. Media were changed by fresh ones containing docetaxel (1 µM or 10 nM) (Sigma-Aldrich, Madrid, Spain), vinorelbine (1 µM or 10 nM) (Sigma-Aldrich, Madrid, Spain) and/or vehicle (ethanol at a final concentration lower than 0.001%). After 3 days, cell viability was measured reading absorbance at 570 nm in a microplate reader (Labsystems Multiskan RC 351, Vienna, VA, USA).

### Measurement of cellular aromatase activity

We analyzed aromatase activity in endothelial cells by the tritiated water release assay, according to the previously described^81^. HUVECs and MCF-7 cells, which have been treated for a week with melatonin 1 mM or its diluent, were cocultured together as indicated previously. Based in previous works^21^, after an homogenous monolayer of preconfluent HUVECs was reached, media were aspirated and changed to serum-free media with the labeled substrate 300 nM [1β-3H(N)]-androst-4-ene-3,17-dione (NEN Life Science Products, Boston, MA, USA) (75–80 Ci/nM) in the presence of docetaxel 1 µM or vinorelbine 1 µM or the diluent (ethanol). Twenty four hours later, flasks were put on ice for 15 min and media were shifted to tubes with 0.25 ml ice-cold 30% tricholoroacetic acid (w/v), vortexed and centrifuged at 1700 *g* for 15 min at 4 °C. The supernatants were extracted with chloroform, vortexed and centrifuged at 1700 g for 15 min at 4 °C. The resulting aqueous supernatants were adsorbed with 5% dextran-coated charcoal (Sigma-Aldrich, Madrid, Spain), vortexed, centrifuged at 1700 g for 15 min at 4 °C and the supernatants were added to vials with scintillation cocktail and counted in a beta counter (Beckman LS 6000 IC, Fulleton, CA, USA). Incubating dishes containing medium with the tritiated androgen, but no cells, we obtained the blank values which were substracted from each sample. The values were also corrected by taking into account the fractional retention of tritium in medium water throughout the processing, which was always higher than 85%.

### Measurement of steroid sulfatase (STS) activity

STS activity in endothelial cells was studied by the formation of estrone from a labelled substrate ([6,7-^3^H(N)]-estrone sulfate ammonium salt)^82^. HUVECs and MCF-7 cells, which have been treated for a week with melatonin 1 mM or its diluent, were cocultured together as indicated previously. As we previously described^83^, when a homogenous monolayer of preconfluent HUVECs was reached, media were changed by fresh ones containing 20 nM [6,7-^3^H(N)]-estrone sulfate ammonium salt (NEN Life Science Products, Boston, MA, USA) (57.3 Ci/mM) in the presence of either docetaxel 1 µM or vinorelbine 1 µM or its diluent (ethanol at a final concentration lower than 0.001%). After 24 h, culture dishes were put on ice for 15 min and 1 ml of the media was placed in tubes containing 5 ml of toluene, vortexed 90 s and centrifuged at 1000 *xg* for 15 min at 4 °C. The resulting organic phase was added to vials with scintillation cocktail and counted in a beta counter. The amount of radioactivity measured in the [^3^H]-toluene was corrected by subtracting the blank values from each sample and by taking into account the fractional retention of tritium in medium water through assay, which was higher than 95%.

### Measurement of 17β-hydroxysteroid dehydrogenase type 1 (17β-HSD1) activity

The activity of 17β-HSD1 was studied in endothelial cells by the formation of estradiol from a labelled substrate [2,4,6,7-^3^H(N)]-estrone^84^. HUVECs and MCF-7 cells, which have been treated for a week with melatonin 1 mM or its diluent, were cocultured together as indicated previously. When we get a homogenous monolayer of preconfluent HUVECs, media were replaced for fresh ones including 2 nM [2,4,6,7-^3^H(N)]-estrone (NEN Life Science Products, Boston, MA, USA) (100 Ci/mM) in the presence of either docetaxel 1 µM or vinorelbine 1 µM or its diluent (ethanol at a final concentration lower than 0.001%) and incubated 30 min. After incubation, media were processed as we previously described^83^, they were transferred to tubes containing 2 ml of diethyl ether, vortexed and centrifuged at 800*xg* for 5 min at room temperature. The aqueous phase was frozen and the resulting organic phase was decanted, evaporated in tubes with 40 µl of estradiol 10 mM. The residue was resuspended in diethyl ether and separated by TLC using dichloromethane/ethyl acetate (4:1; v/v). Once the spots had been visualised, excised and eluted with methanol, they were counted in a liquid scintillation counter. Values were corrected for blanks and tritium recovery, which was higher than 85%.

### Human angiogenesis RT^2^ profiler PCR array

Pathway-focused gene expression profiling was performed using a 96-well Human Angiogenesis RT^2^ Profiler PCR Array (Qiagen, USA). In this array, each well contains all the components required and designed to generate single, gene-specific amplicons, testing the expression of 84 genes related to angiogenesis (growth factors and receptors, adhesion molecules, proteases, inhibitors and other matrix proteins, transcription factors, cytokines and chemokines), plus 5 housekeeping genes. Each RT^2^ Profiler PCR array plate also includes controls for data normalization, genomic DNA contamination detection, RNA sample quality and general PCR performance. As in previous works^34^, HUVECs, which have been treated for a week with melatonin 1 mM, were cultured into 60 × 15 mm plates in VCBM supplemented with 2% FBS and incubated at 37 °C for 24 h to allow for cell attachment. Then media were replaced by fresh ones with 2% FBS and containing either docetaxel (1 µM) or vinorelbine (1 µM) alone or in combination with 1 mM melatonin and/or vehicle (ethanol at a final concentration lower than 0.0001%). After 4 h of incubation, total RNA was extracted and reverse transcribed. The cDNA template was mixed with the appropriate amount of RT^2^ SYBR Green qPCR Mastermix (Qiagen GmbH, Germany), aliquoted 25 µl to each well of the same plate, and then the real-time PCR cycling program was performed in an MX3005P (Agilent, CA, USA) following the manufacturer’s instructions. Amplification was initiated by 1 cycle at 95 °C for 10 min and then performed for 40 cycles for quantitative analysis using the following temperature profile: 95 °C for 30 sec (denaturation); 60 °C for 60 sec (annealing/extension). Dissociation curves were performed to verify that only a single product was amplified. The Ct data for each gene were analyzed using the Qiagen RT^2^ profiler PCR array data analysis software. Data are represented as fold-regulation between the experimental groups and the control cells. Fold-change values less than one indicate a negative or downregulation, and the fold-regulation is the negative inverse of the fold-change.

### Measurement of specific mRNA gene expression

Measurement of different mRNA genes expression in endothelial cells were performed by qRT-PCR after incubation of cells, which have been treated for a week with melatonin 1 mM, with either docetaxel 1 µM or vinorelbine 1 µM or its diluent (ethanol at a final concentration lower than 0.001%) for 4 h. The total cellular RNA was isolated from HUVECs and purified with the Nucleospin RNA II Kit (Machenery-Nagel, Düren, Germany) following the manufacturer’s instructions. As we previously described^83^, integrity of RNA was evaluated by electrophoresis in ethidium bromide-stained 1% agarose-Tris-borate EDTA gels. The absorbance ratio A_260 nm_/A_280 nm_ was> 1.8. For cDNA synthesis 1 µg of total RNA was denatured at 65 °C for 10 min and reverse transcribed for 50 min at 45 °C with cDNA Synthesis kit (Bioline, London, UK) in a final volume of 20 μl in the presence of 500 ng of oligo (dT) 12–18 primers. The primers used for amplification (Sigma Genosys Ltd., Cambridge, UK) are listed in Table 1. As a control quantification, s14 mRNA was also subjected to real-time RT-PCR using a set of specific primers (Sigma Genosys Ltd., Cambridge, UK). RT-PCRs were performed in a MX3005P system (Stratagene, La Jolla, CA, USA) using Brilliant SYBR Green PCR Master Mix (Applied Biosystems, Madrid, Spain) following the manufacturer’s instructions. Amplifications were performed for 40 cycles using the following temperature profile: 60 °C, 45 sec (annealing); 72 °C, 30 sec (extension) and 95 °C, 30 sec (denaturation). Each reaction was run ninefold by quadruplicate. Melting curves were performed to verify that only a single product with no primer-dimers was amplified. For the primers used there were no differences between transcription efficiencies, and the fold-change in each sample was calculated by the 2^−∆∆Ct^ method^85^. The fractional cycle at which the amount of amplified targets becomes significant (Ct) was automatically calculated by the PCR program.

**Table 1:** Primers used for amplification of mRNA transcripts.

Human Gene	Sense primer (5′-3′)	Antisense primer (5′-3′)
*17β-HSD1*	CTTATGCGAGAGTCTGGCGGTTC	GGTATTGGTAGAAGCGGTGGAAGG
*AKT1*	AAGTACTCTTTCCAGACCC	TTCTCCAGCTTGAGGTC
*ANG1*	GAAGGGAACCGAGCCTATTC	AGGGCACATTTGCACATACA
*ANGPT2*	AAGAGAAAGATCAGCTACAGG	CCTTAGAGTTTGATGTGGAC
*ANPEP*	CCTTCATTGTCAGTGAGTTC	CAGCAAAGAAGTTAAGGATGG
*CCL2*	AGACTAACCCAGAAACATCC	ATTGATTGCATCTGGCTG
*CYP19A1*	GTCGTGGACTTGGTCATGC	CGAGTCTGTGCATCCTTCC
*CXCL6*	CCTCTCTTGACCACTATGAG	GTTTTGGGGTTTACTCTCAG
*FIGF*	GCTCTTTGAGATATCAGTGC	CACTTACAACCTGTATGATTGG
*FGFR3*	GAAGATGCTGAAAGACGATG	GCAGGTTGATGATGTTTTTG
*HIF-1α*	AGCCGAGGAAGAACTATGAACATAA	GTGGCCTGTGCAGTGCAA
*IGF1*	CCCAGAAGGAAGTACATTTG	GTTTAACAGGTAACTCGTGC
*JAG1*	ACTACTACTATGGCTTTGGC	ATAGCTCTGTTACATTCGGG
*LECT1*	GAAATCCAGAGGGAAAGAAG	GGTCTGGTTTCATTATTCAGTC
*MMP-14*	GATAAACCCAAAAACCCCAC	CTCCTTGAAGACAAACATCTC
*NOS3*	CAACCCCAAGACCTACG	CGCAGACAAACATGTGG
*Cyp19A1 pI.7*	AACACTCAGCTTTTTCCCAAC	CTTGCTGATTTCACCCCTTT
*STS*	TCCGTTCCTGCTTGTCTTGTC	CCTGGTCCGATGTGAAGTAGATG
*Tie2*	AAGACCTACGTGAATACCAC	GAAACAGAGGGTATACAGATG
*TIMP3*	CATGTGCAGTACATCCATAC	AGGTGATACCGATAGTTCAG
*VEGF-A*	TGGTGATGTTGGACTCCTCA	GGGCAGAATCATCACGAAGT
*VEGF-B*	GAAAGTGGTGTCATGGATAG	ATGAGCTCCACAGTCAAG
*VEGF-C*	GTGTCCAGTGTAGATGACTC	ATCTGTAGACGGACACACATG
*VEGFR-1*	CCTTGAACACAGCTCAAGCA	CCCAGATTATGCGTTTTCCA
*VEGFR-2*	GTACATAGTTGTCGTTGTAGG	TCAATCCCCACATTTAGTTC
*VEGFR-3*	AGGTATTACAACTGGGTGTC	TTCCTCAAATGTCTTCATCC
*S14*	TCACCGCCCTACACATCAAAC	TCCTGCGAGTGCTGTCAGAG

### Endothelial cell migration assay: wound healing assay

HUVEC control or melatonin 1 mM, treated for a week, cells were cultured into 6-well plates (Falcon) in VCBM enriched with 2% FBS and were allowed to reach full confluence. Based in previous works^23^, a line of cells was scraped away in each plate using a pipette tip. Cells were washed with PBS and docetaxel 10 nM or vinorelbine 10 nM or its diluent (ethanol at a final concentration lower than 0.001%) was added to each plate. After that, four randomly selected views along the scraped line in each plate were photographed using an ORCA R2 camera (Hamamatsu Photonics, Massy Cedex, France) attached to a microscope set Nikon Ti (Werfen Group, Barcelona, Spain) at 10x magnification. Photomicrographs were taken every 10 minutes until the wound was completely filled in controls (after 10 hours). Initial and final wound sizes were determined using the Nis Elements v.3.8 software (Nikon, Tokyo, Japan) and the difference between the two was used to determine migration distance using the following formula: initial wound size minus final wound size divided by two.

### Endothelial cell differentiation assay: endothelial cell capillary-like tube formation assay

To study HUVECs tube formation we used the *In vitro* Angiogenesis Assay Tube Formation Kit (Cultrex, Trevigen Inc, Gaithersburg, MD, USA) following the manufacturer’s instructions. As we previously described^24^, growth factor-reduced Basement Membrane Extract (BME) was added into a 24-well plate and polymerized for 1 h at 37 °C. After that, cells (3 × 10^5^ per well), treated or not with melatonin 1 mM for a week, were cultivated in VCBM with 2% FBS in the presence of either docetaxel 10 nM or vinorelbine 10 nM or its diluent (ethanol at a final concentration lower than 0.001%). After 4 h, 5 random fields were selected per well and tubular structures were photographed with a camera Nikon Sight DS-SML1 (Sendai Nikon Corporation, Miyagi, Japan) attached to a fluorescence microscope Nikon Eclipse TS100 (Kurobane Nikon Co Ltd, Tochigi, Japan) at 4×. The length of the tubular network was measured using ImageJ 1.45 S software. We repeat this assay four times.

### Western blotting

Cells were washed with PBS and proteins were collected using RIPA buffer, containing 1% protease inhibitor cocktail. The protein concentration was measured using the method of Lowry^86^ in the spectrophotometer (Multiskan ex, Thermo Scientific) at a wavelength of 620 nm, using a standard curve of albumin. As in previous works^87^, cellular proteins were separated by SDS-PAGE and transferred to a polyvinylidene fluoride (PVDF) membrane (Bio-Rad) from the polyacrylamide gel. Then, the membranes were blocked by incubation (1 hour at room temperature) in TBS-T buffer (10 mM Tris-HCl, pH 7.6, 150 mM NaCl, 0.05% Tween 20) with 3% bovine serum albumin (BSA). Later the membranes were incubated with the primary antibody, first for 1 h at room temperature and then overnight at 4 °C and under stirring, and then incubated with the respective fluorescent secondary antibody for 30 minutes at room temperature. We used the following antibodies: rabbit anti-AKT antibody (Cell Signaling Technology, Danvers, MA, USA), rabbit anti-P-AKT antibody (Santa Cruz, CA, USA), mouse anti-ERK½ antibody (Santa Cruz, CA, USA), rabbit anti-P-ERK½ antibody (Cell Signaling Technology, Danvers, MA, USA), mouse anti-GAPDH antibody (Santa Cruz, CA, USA). Protein bands were detected by incubating with anti-rabbit IRDye- 680RD LI-COR Biotechnology, Lincoln, Nebraska, USA Red (700 nm) or anti-mouse IRDye-800CW LI-COR Biotechnology, Lincoln, Nebraska, USA Green (800 nm). Fluorescence was detected using the LI-COR Odyssey IR Imaging System V3.0 system (LI-COR Odyssey Biosciences). The optical density of the bands was determined with the ImageJ program.

### Vascular permeability assay

Endothelial permeability was determined by diffusing of 40 kDa fluorescein isothiocyanate (FITC)-dextran through the endothelial cells^88^. As in previous works^87^, endothelial cells, control and melatonin treated for a week, were grown to confluence on collagen-coated inserts. In order to stimulate endothelial cell permeability VEGF 4 ng/ml was added to the plates and inserts and docetaxel 10 nM, vinorelbine 10 nM and/or melatonin 1 mM were added. After 24 h, FITC-dextran solution was added to the cultures. The fluorescence intensity of FITC-dextran that crossed the endothelial monolayer at 30, 60 and 90 min was measured by using a microplate reader at 485/530 nm (Labsystems Multiskan RC 351, Vienna, VA, USA).

### Immunofluorescence assay

Immunolabeling was analyzed in control or melatonin treated HUVECs grown on coverslips as described previously^87^. Cells were treated with docetaxel 10 nM, vinorelbine 10 nM and/or melatonin 1 mM. After 24 h, cells were fixed in 100% methanol (5 min) at room temperature. Then, after washing carefully, crystals were placed in a Petri dish and cells were permeabilized with PBS with 10% goat serum, 0.3 M glycine, 1% BSA and 0.1% tween for 1 h at room temperature. Primary antibody staining was performed for rabbit policlonal anti-VE-cadherin (ab33168) (Abcam, Cambridge, UK) at 1 µg/ml. After several PBS washes, cells were incubated for 1 h at room temperature with the secondary antibody conjugated with a fluorochrome, FITC (Jackson ImmunoResearch Laboratories, Inc., USA). Then, crystals were washed with 1x PBS, dried thoroughly and a drop of vectashield mounting medium (Vector Laboratories, Peterborough, UK) was added. Observation was made with a LSM51O laser confocal microscope (Zeiss).

### Chick chorioallantoic membrane model of angiogenesis

As an *in vivo* model for studying angiogenesis we used the chick chorioallantoic membrane (CAM) model assay as described elsewhere^88^. All experiments were approved by the Bioethics Committee of the University of Cantabria and carried out in full accordance with the guidelines of the European Community (2010/63/EU) and the Spanish regulations (RD 53/2013). Fertilized eggs were incubated at 37 °C in a humidified incubator for 3 days. To allow detachment of the developing CAM shell we removed 4 ml of egg albumin with a hypodermic needle. At day 4, the shells were covered with a transparent adhesive tape and a small window sawed with scissors on the broad side directly over the avascular portion of the embryonic membrane. At day 11, alginate beads containing PBS, VEGF, docetaxel 1 μM, vinorelbine 1 μM and/or melatonin 1 mM per bead were grafted on the CAM. VEGF 1.57 pM was used as a positive control of angiogenic compound whereas PBS was used as a negative control. Chemical agents were dissolved in 1% dimethyl sulfoxide. After 72 h new blood vessels converging towards the implant were counted at 5 x magnification under a STEMI SR stereomicroscope equipped with a 100 mm objective with adapter ring 47070 (Zeiss) and fixed with 7% buffered formalin and photographed.

### Statistical analysis

Using GraphPad Prism software, statistical differences between groups were analyzed by one way analysis of variance (ANOVA), followed by the Student-Newman-Keuls test. Results were considered as statistically significant at p < 0.05. The data are showed as the mean ± standard errors of the mean (S.E.M.).

## Supplementary information

Supplementary information.
