# Supplementary material for: Usefulness of melatonin as complementary to chemotherapeutic agents at different stages of the angiogenic process
Source: Sci Rep. 2020 Mar 16;10:4790. doi: 10.1038/s41598-020-61622-x (PMC7076026; doi:10.1038/s41598-020-61622-x)
Supplement: Supplementary file 1 — Supplementary information. [file 41598_2020_61622_MOESM1_ESM.pdf]

Usefulness of melatonin as complementary to chemotherapeutic agents at different stages of the angiogenic process

Authors and affiliations:

Alicia González-González[1], Alicia González[1*], Noemi Rueda[1], Carolina Alonso-González[1], Javier Menéndez Menéndez[1], Carlos Martínez-Campa[1*], Stefania Mitola[2], Samuel Cos[1]

[1] Department of Physiology and Pharmacology, School of Medicine, University of Cantabria and Instituto de Investigación Sanitaria Valdecilla (IDIVAL), 39011 Santander, Spain

[2] Department of Molecular and Translational Medicine, Laboratory for Preventive and Personalized Medicine, University of Brescia, 25123 Brescia, Italy

*** Main corresponding author:** Dr. Alicia González, Department of Physiology and Pharmacology, School of Medicine, University of Cantabria, Cardenal Herrera Oria s/n, 39011 Santander, Spain. Telephone number: 34 942 201965; Fax number: 34 942 201903. E-mail: gonzalav@unican.es.

Additional corresponding author: Dr. Carlos Martínez-Campa, Department of Physiology and Pharmacology, School of Medicine, University of Cantabria, Cardenal Herrera Oria s/n, 39011 Santander, Spain. Telephone number: 34 942 201963; Fax number: 34 942 201903. E-mail: martinezcm@unican.es

Supplementary excel file with the expression changes of the 84 genes studied

FOLD REGULATION

	C-M		C-D		D-DM		C-V		V-VM
CXCL10	-11,637	CXCL10	-19,222	FIGF	-31,559	CXCL10	-29,995	MMP14	-18,328
EGF	-4,8872	CCL2	-16,734	TIMP1	-21,706	IL6	-7,9447	NOTCH4	-12,871
CXCL8	-4,8422	TGFB2	-8,1946	ANPEP	-11,632	CCL2	-6,5887	COL18A1	-9,1641
CCL11	-4,425	CXCL1	-5,8213	EFNA1	-11,314	PF4	-6,5735	ERBB2	-7,6529
COL4A3	-4,425	IL6	-2,7985	NOS3	-10,778	CXCL9	-6,5432	NOS3	-7,2904
IFNG	-4,425	PF4	-2,378	PLAU	-9,5798	IFNG	-6,0909	TIMP1	-6,7552
IGF1	-4,425	PTGS1	-2,123	ANGPT1	-9,3179	LECT1	-6,0909	TYMP	-6,0461
IL1B	-4,425	ANGPTL4	-1,879	EFNB2	-9,2535	PROK2	-6,0909	SERPINF1	-5,6412
LECT1	-4,425	CTGF	-1,596	ADGRB1	-7,7275	TNF	-6,0909	PTGS1	-5,5251
PLG	-4,425	FGF1	-1,3485	NOTCH4	-7,4643	CCL11	-6,0909	ANGPT1	-5,4114
PROK2	-4,425	PDGFA	-1,0556	TEK	-7,4127	CXCL1	-6,0071	MDK	-4,1583
TNF	-4,425	VEGFA	-1,041	EPHB4	-7,0128	COL4A3	-4,5842	ANGPTL4	-3,9889
CXCL1	-4,2644	MMP9	-1,0243	MDK	-6,8685	HGF	-3,4742	TIE1	-3,8264
F3	-3,9788	CXCL6	-1,0149	ERBB2	-6,774	THBS2	-3,3404	TGFA	-3,8
IFNA1	-3,9605	EFNA1	1,0177	ANGPTL4	-6,5432	PLG	-3,1675	SERPINE1	-3,7218
CXCL6	-3,4518	FGF2	1,0634	NRP2	-6,5432	EGF	-3,0525	ANPEP	-3,4011
CXCL9	-3,4082	EDN1	1,0658	ENG	-6,2333	TGFB2	-2,8745	VEGFB	-3,3776
HGF	-3,3613	TIMP3	1,0857	AKT1	-6,021	MDK	-2,3403	EFNA1	-3,2625
PF4	-2,967	MDK	1,161	SPHK1	-5,9381	IFNA1	-2,1705	PLAU	-3,2625
FGFR3	-2,5979	CXCL5	1,1909	FN1	-5,6569	VEGFA	-2,206	SPHK1	-3,2176
IL6	-2,3795	CXCL9	1,2075	MMP2	-5,5022	IL1B	-2,125	KDR	-3,1514
FGF1	-2,162	SERPINE1	1,2187	TGFB1	-5,4642	LEP	-2,0125	HPSE	-2,9404
ANGPT2	-2,1297	VEGFC	1,2501	COL18A1	-5,3517	HIF1A	-1,5547	EFNB2	-2,9201
VEGFC	-2,1273	THBS1	1,3336	COL4A3	-5,3147	CTGF	-1,2805	TEK	-2,8206
CXCL5	-2,0548	LEP	1,3367	CDH5	-5,2416	TIMP3	-1,2716	TGFB1	-2,8206
LEP	-1,8821	THBS2	1,3648	KDR	-5,2416	PECAM1	-1,107	CDH5	-2,5597
EFNB2	-1,7847	EGF	1,426	S1PR1	-5,1694	FIGF	-1,1019	AKT1	-2,4217
MMP9	-1,7601	HGF	1,426	MMP14	-5,0982	MMP9	1,0305	THBS1	-2,307
ITGAV	-1,7581	NOTCH4	1,5569	FLT1	-5,0281	FGF2	1,0473	PDGFA	-2,2595
TGFB2	-1,7299	COL18A1	1,5605	SERPINF1	-4,9588	IGF1	1,0546	ANG	-2,1825
ID1	-1,6923	CCL11	1,575	TIE1	-4,4691	ADGRB1	1,0668	CXCL5	-2,162
FGF2	-1,6047	IFNA1	1,575	TIMP2	-4,4076	ANGPT1	1,0817	NRP2	-2,1597
NOS3	-1,5394	IFNG	1,575	F3	-4,3169	EDN1	1,0892	TIMP2	-2,1373
ANGPT1	-1,5234	IGF1	1,575	ANG	-4,1699	TIMP2	1,0994	ENG	-2,1248
ITGB3	-1,5077	IL1B	1,575	NRP1	-4,1125	PTGS1	1,1173	S1PR1	-2,1153
COL18A1	-1,4835	LECT1	1,575	PDGFA	-4,084	ANG	1,1199	EPHB4	-2,1081
HPSE	-1,4614	PLG	1,575	TYMP	-4,0558	CXCL6	1,366	MMP2	-2,0851
SERPINF1	-1,4446	PROK2	1,575	ITGAV	-3,8106	THBS1	1,3787	FN1	-2,0605
ADGRB1	-1,3762	TNF	1,575	FGFR3	-3,6301	CXCL5	1,4273	CXCL1	-2,0605
PDGFA	-1,3186	CXCL8	1,6419	THBS2	-3,5801	ITGB3	1,4675	CXCL6	-2,0063
TGFBR1	-1,308	TGFB1	1,876	ITGB3	-3,4343	ANGPTL4	1,5018	FGFR3	-1,9562
CTGF	-1,2855	ANG	1,925	PTGS1	-3,4343	ANPEP	1,5227	TGFB2	-1,8867
JAG1	-1,2737	TIMP2	2,0916	CCL11	-3,2944	VEGFB	1,5227	NRP1	-1,6757
FN1	-1,2576	S1PR1	2,2069	CXCL9	-3,2944	FN1	1,644	PGF	-1,5757
VEGFA	-1,2475	HIF1A	2,2377	EGF	-3,2944	EFNA1	1,6547	LEP	-1,3449
ANPEP	-1,1734	TIE1	2,2637	IFNA1	-3,2944	S1PR1	1,7619	IL6	-1,2363

PLAU	-1,1653	TGFBR1	2,2953	IFNG	-3,2944	TIE1	1,8021	F3	-1,1942
AKT1	-1,1572	ITGAV	2,3006	IL1B	-3,2944	NOTCH4	1,9133	FGF2	-1,1297
THBS1	-1,1532	PLAU	2,3544	LECT1	-3,2944	FLT1	2,0123	EDN1	-1,0324
PECAM1	-1,1479	PGF	2,4261	MMP9	-3,2944	VEGFC	2,0123	CXCL10	-1,0253
EPHB4	-1,1439	VEGFB	2,46	PF4	-3,2944	TGFB1	2,0312	ITGAV	1,0898
NOTCH4	-1,1308	COL4A3	2,5409	PLG	-3,2944	FGF1	2,0456	HIF1A	1,1925
FLT1	-1,1101	FN1	2,5409	PROK2	-3,2944	MMP2	2,0456	FLT1	1,575
SPHK1	-1,1062	SPHK1	2,6672	TNF	-3,2944	ITGAV	2,0689	MMP9	1,925
TEK	-1,0999	PECAM1	2,6796	SERPINE1	-3,2266	ANGPT2	2,0715	ID1	2,0916
HIF1A	-1,086	ANGPT1	2,8258	HPSE	-3,1167	COL18A1	2,0821	CCL2	2,2069
MDK	-1,0538	EFNB2	2,8586	JAG1	-3,0314	NRP1	2,0963	ITGB3	2,2953
THBS2	-1,0191	CDH5	2,8785	TGFBR1	-2,9282	HPSE	2,0978	TGFBR1	2,3006
MMP2	-1,0039	ANPEP	2,8986	LEP	-2,9079	NRP2	2,1113	PECAM1	2,3544
TIMP1	1,0123	AKT1	3,0216	HIF1A	-2,6759	EPHB4	2,1192	ADGRB1	2,4261
S1PR1	1,036	ITGB3	3,0286	VEGFB	-2,6574	NOS3	2,1538	FGF1	2,574
TIE1	1,0372	MMP2	3,0427	FGF2	-2,639	TGFBR1	2,1936	CTGF	2,6281
VEGFB	1,0553	FLT1	3,0851	PGF	-2,3457	TYMP	2,214	CXCL8	2,856
PGF	1,059	SERPINF1	3,1282	ID1	-2,2974	CDH5	2,4396	THBS2	3,4438
CCL2	1,075	HPSE	3,2236	IL6	-2,2815	AKT1	2,4623	IL1B	3,5162
KDR	1,1142	EPHB4	3,345	THBS1	-1,981	SERPINE1	2,5198	VEGFA	3,6402
ANG	1,1429	TYMP	3,439	EDN1	-1,976	PDGFA	2,5787	PF4	3,9286
TGFB1	1,1535	ADGRB1	3,6944	HGF	-1,9625	SERPINF1	2,6512	EGF	4,1526
EDN1	1,1696	MMP14	3,7633	CXCL5	-1,9588	TEK	2,7195	PLG	5,2198
CDH5	1,1764	ERBB2	3,772	PECAM1	-1,9319	ID1	2,9147	VEGFC	5,5174
ENG	1,1955	ENG	4,2242	IGF1	-1,8921	ERBB2	3,1383	JAG1	5,996
EFNA1	1,2053	TEK	4,2831	ANGPT2	-1,6702	KDR	3,605	IFNA1	6,8876
MMP14	1,215	ANGPT2	4,7415	TGFA	-1,6702	MMP14	3,6723	ANGPT2	7,1801
NRP1	1,2449	NRP2	4,8077	VEGFA	-1,5476	ENG	3,7755	IGF1	7,8571
TYMP	1,2449	ID1	4,8299	FGF1	-1,434	JAG1	3,793	FIGF	8,1342
TIMP3	1,3037	JAG1	4,8973	CXCL6	-1,3566	PLAU	4,0093	HGF	8,9631
TIMP2	1,3482	NOS3	5,8509	VEGFC	-1,2483	PGF	4,084	CCL11	9,0255
ERBB2	1,3607	NRP1	5,8645	TIMP3	-1,0425	TIMP1	4,5736	CXCL9	9,1514
NRP2	1,3654	F3	6,5372	CXCL1	1,4241	TGFA	4,7678	IFNG	9,1514
ANGPTL4	1,3941	KDR	7,22	CTGF	1,8411	CXCL8	4,8232	LECT1	9,1514
SERPINE1	1,4483	FGFR3	7,3039	CXCL8	2,098	EFNB2	6,5584	PROK2	9,1514
PTGS1	2,0519	FIGF	7,5266	CXCL10	2,1886	SPHK1	6,6192	TNF	9,1514
FIGF	2,1786	TGFA	9,3524	TGFB2	2,969	F3	8,0371	TIMP3	11,3451
TGFA	2,7786	TIMP1	11,461	CCL2	4,7899	FGFR3	10,0794	COL4A3	19,2129

Figure S 9

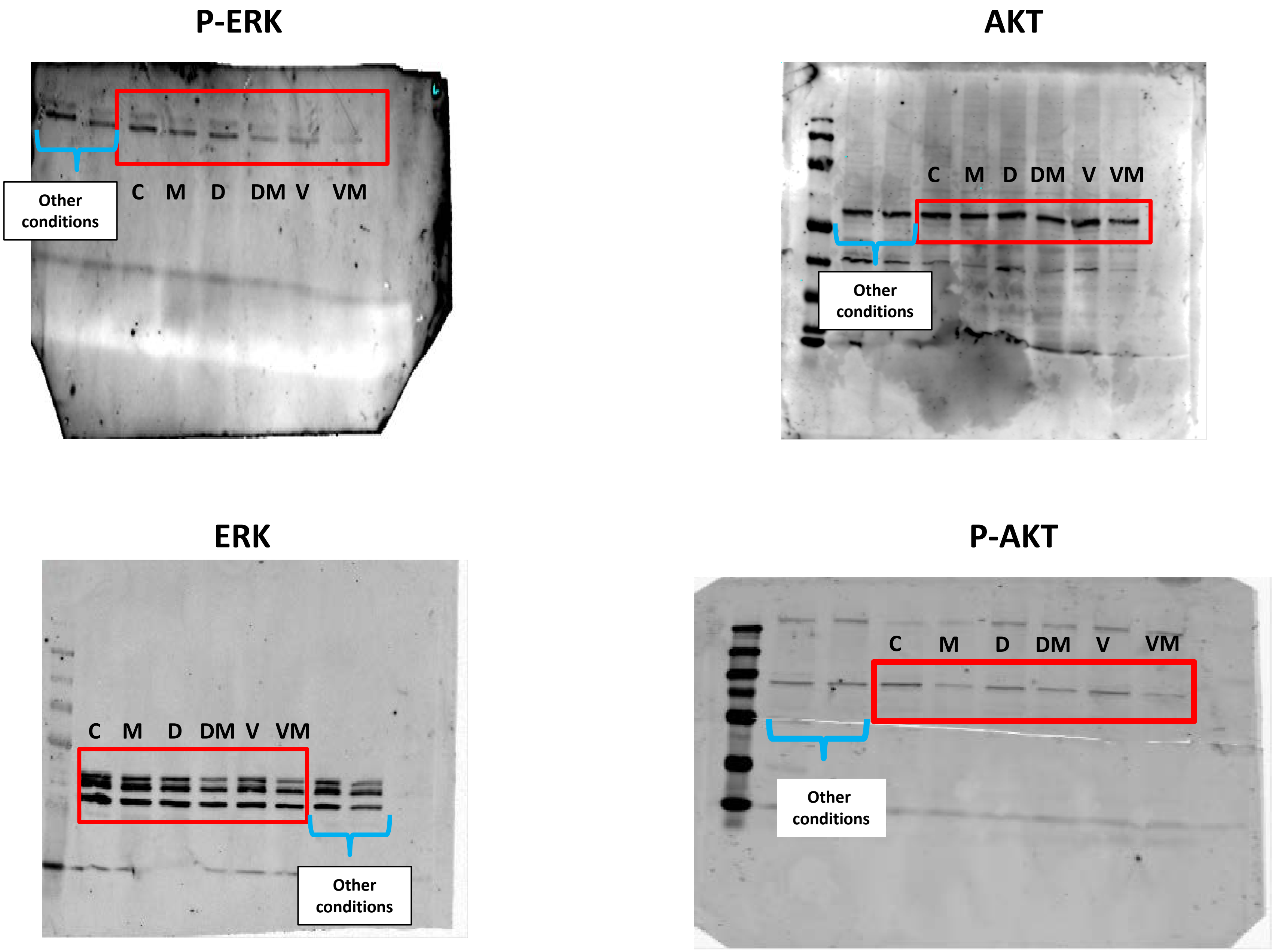

P-ERK
Other conditions
C M D DM V VM
AKT
C M D DM V VM
Other conditions
ERK
C M D DM V VM
Other conditions
P-AKT
C M D DM V VM
Other conditions
